# Collaborating with transgender youth to educate healthcare trainees and professionals: randomized controlled trial of a didactic enhanced by brief videos

**DOI:** 10.1186/s12889-022-14791-5

**Published:** 2022-12-26

**Authors:** Andrés Martin, Jillian Celentano, Christy Olezeski, Justin Halloran, Brent Penque, Jemel Aguilar, Doron Amsalem

**Affiliations:** 1 Yale Child Study Center, Yale School of Medicine, 230 South Frontage Road, New Haven, CT 06520-7900 USA; 2 Simulated Participated Program, Teaching and Learning Center, Yale School of Medicine, New Haven, CT USA; 3 Department of Social Work and Marriage and Family Therapy, Southern Connecticut State University, New Haven, CT USA; 4 Gender Clinic, Departments of Pediatrics and Psychiatry, Yale School of Medicine, New Haven, CT USA; 5 Department of Psychiatry, Columbia University Vagelos College of Physicians and Surgeons, New York, NY USA

**Keywords:** Transgender, Gender-diverse, Adolescents, Health, Mental health, Health education

## Abstract

**Background:**

In collaboration with members of the transgender and gender diverse (TGD) community, we created a didactic resource about the unique needs of TGD youth.

**Methods:**

We developed teaching materials enhanced by video clips of two TGD adolescents openly sharing aspects of their lived experience. We compared the video and no video conditions in a randomized controlled trial (RCT) in which participants were assigned to one of four parallel conditions: 1) a transgender [TgV] or 2) a cisgender [CgV] woman presenting with videos embedded into the presentation, 3) the same cisgender woman presenting without the videos [CgN], or 4) a no intervention control [NiC]. Our primary outcome was change in the total score of the Transgender Knowledge, Attitudes, and Beliefs Scale (T-KAB).

**Results:**

We recruited and proportionally randomized 467 individuals, 200 of whom completed ratings before and after the intervention: TgV (*n* = 46), CgV (*N* = 46), CgN (*n* = 44), and NiC (*n* = 64). Mean scores on all measures of TGD acceptance increased in the video group, compared to the no video group. Improvements persisted after 30 days (*p* < 0.01), except on perceptions about TGD family members. The three active intervention groups did not differ in efficacy.

**Conclusions:**

These findings provide empirical evidence that a well-informed presenter, regardless of their gender, can achieve similar improvements in perceptions and knowledge about TGD youth when using a resource that can be disseminated free of cost.

**Supplementary Information:**

The online version contains supplementary material available at 10.1186/s12889-022-14791-5.

## Introduction

Transgender and gender diverse (TGD) youth are an increasingly visible yet often poorly understood segment of the population [1]. Changing social mores and a growing understanding of gender identity as a normative continuum rather than a pathological condition have opened opportunities to improve the lives of children and adolescents on unique paths toward their gendered selves. The stakes are high: disparities and social injustices are daily realities for many, if not most, TGD youth facing unique burdens in addition to the already significant challenges of adolescence and identity formation. The minority stress model [2–4] provides a useful framework through which to understand the threats toward reaching optimal developmental outcomes for TGD youth. *Distal*, or external factors such as non-acceptance, social exclusion, bullying, or violence can have significant impacts on health and education. *Proximal*, or internal factors can include distress regarding a misalignment between physical sex characteristics and gender identity, or internalized transphobia that can result from living in an inhospitable environment in which a core aspect of one’s individuality is regularly questioned or challenged [5].

All members of society, and particularly their healthcare providers, can have considerable influence on the lives of TGD youth. Well-meaning individuals often lack the knowledge or skills to interact unobtrusively with TGD youth. Children and adolescents who feel misunderstood, judged, or maligned are less likely to share their concerns and be open with clinicians or other adults important in their lives. The consequences of such withdrawal can be devastating, as reflected by the disproportionately higher rates of self-harm and suicidal behavior among TGD youth when compared to rates in their cisgender peers [6, 7]. Alternatively, settings that are welcoming, and providers who are accepting, non-judgmental, supportive, and informed about best evidence-based practices, are not just equitable and patient-centered: they lead to improved health outcomes [8].

Training activities that increase knowledge, skills, and attitudes are central in moving toward care that is informed about gender diversity. Education materials based on best current practices, contemporary pedagogy, and use of video can facilitate the diffusion of relevant content [9, 10]. We sought to build on those efforts and to narrow the gap in training among healthcare providers [11] by partnering with members of the TGD community in creating updated resources.

Developing readily accessible materials with the buy-in, and indeed with the active collaboration of members of the TGD community, can result in information that stays true to the needs of those most directly impacted by it. In becoming co-creators of such resources, TGD individuals not only enhance the legitimacy of the relevant content but join in the shared task of improving the training of healthcare trainees and professionals. Such an approach abides by the dictum of “nothing about us without us” [12, 13] but does not exclusively relegate the minoritized group with the task of educating others. As aptly put in the first published book on pediatric gender identity ([14], p. 4), “…the onus of understanding…rests not with the patient but with the clinician. This allows the youth to remain in the role of the cared-for patient, rather than caring for the provider.”

In collaboration with members of the TGD community and with content experts in the field, we created a didactic resource about the unique healthcare needs of TGD youth. Central to these materials are video clips of two TGD adolescents openly sharing aspects of their lived experience. We go on to describe these materials and how we tested their efficacy in a randomized controlled trial (RCT).

## Methods

### Didactic materials

We edited video excerpts from professionally filmed interviews of two 17-year-old transgender adolescents, one female (“Monica”) and one male (“Parker”). One of the co-authors, a cisgender woman (CO) conducted the interviews, and another, a transgender woman (JC), supported the youth during the several takes that were required. Both women have over 15 years of experience in transgender health, education, and training. The video components sought to reflect the realities, both objective and subjective, of being transgender in a way that was realistic and avoided performative dramatization that could entrench prevailing stereotypes. They were designed for a lay audience rather than for specialists in transgender health care. The resulting ten clips have median [range] and cumulative durations of 2″15 [57′ – 2″12] and 15 min, respectively. The clips exemplify and complement key aspects of the didactic relevant to the routine healthcare needs of TGD youth, including introductions and 1) gender exploration; 2) gender identity vs. sexual orientation; 3) names and pronouns; 4) the impact of using the wrong pronouns; 5) gender dysphoria; 6) gender and mental health; 7) gender joy; and 8) advice for the novice. The video clips are free for viewing or download and can be accessed at https://figshare.com/s/20c965b683cae24648da.

### Study design

We conducted a RCT in which participants were assigned in equal parts to one of four parallel conditions: 1) a transgender woman presenting with videos embedded into the teaching materials [TgV]; 2) a cisgender woman presenting with embedded videos [CgV]; 3) the same cisgender woman presenting with no embedded videos [CgN]; or 4) a no intervention control [NiC]. Whether embedded or shown afterward, the same video clips were presented as part of a 45-min didactic. Participants completed a baseline assessment before their respective intervention (PRE). For the no video conditions (CN, NiC), we showed the same videos only after collecting outcome measures post-intervention (POST). This design permitted us to make comparisons across video use (yes / no), type of presenter (trans- / cis- gender), and the interaction of the two.

We delivered didactic content using pre-recorded materials, followed by a live question and answer period with the two presenters. We opted for a pre-recorded webinar format using Zoom (San Jose, CA) for two reasons: 1) to ensure reproducibility of content, and specifically for the TgV intervention, which was shown to both the TgV and NiC groups; and 2) to prevent “Zoom bombing”, which could potentially occur while addressing a topic that remains controversial for some.

### Ethics approval and participant recruitment

We registered the study in ClincialTrials.org (NCT05080335), and after obtaining approval from the institutional review boards (IRBs) of Yale University (HIC # 2000031117) and Southern Connecticut State University (Protocol # 535), we offered enrollment via email. The ethical approval and consent to participate from both IRBs were in accordance with the relevant guidelines and regulations set forth in the Declaration of Helsinki. Participants were adult volunteers 18 years or older, drawn as a convenience sample from several professional sources and training levels: 1) members of the Yale Child Study Center (YCSC) community; 2) undergraduate students from Southern Connecticut State University (SCSU); 3) students from the Graduate Entry Program in Nursing (GEPN) at the Yale School of Nursing (YSN); 4) students from the Yale Physician Assistant online program; 5) medical students participating in a national mentorship network supported by the Klingenstein Third Generation Foundation (KTGF, [15, 16]); and 6) members of the American Academy of Child and Adolescent Psychiatry (AACAP) affiliated with its Alliance for Learning Innovation (AALI). The only identifying information we collected, email addresses, were used for the sole purpose of inviting and scheduling participation. We maintained these personal data in a secure file accessible only to the PI and destroyed it upon study completion.

All participants provided written informed consent before joining the educational session, which was an optional enrichment activity, typically offered through interest groups and elective courses, and not as part of required coursework. As such, no class time was used to conduct the study, which took place over the course of four early evenings (5:30–7 PM) between January and March of 2022. Participation required 90 min of time and posed minimal survey burden, with an estimated duration of less than 5 min for PRE and POST assessments each, for a total of under 10 min.

### Outcome measures

Our primary outcome was change in the total score of the Transgender Knowledge, Attitudes, and Beliefs Scale (T-KAB) [17]. The T-KAB consists of 22 items, divided into three subscales: 1) Social tolerance (ST, 7 items); 2) Comfort and contact (CC, 7 items); and 3) Acceptance of the gender spectrum (ACC, 8 items), which are added into a total score (TOT). Some of the items are reverse scored to prevent acquiescence bias. To provide greater gradation in response, we adjusted the original 4-point ordinal scale (strongly disagree = 1; disagree = 2; agree = 3; and strongly agree = 4) into a 6-point scale (by adding the midpoints of “somewhat disagree” and “somewhat agree.”) The resulting values ranged from 1 to 6 for individual items; 7–42 for ST and CC; 8–22 for ACC; and 22–132 for the overall score. Of note, neither the original nor the adjusted anchor points include a neutral option (“neither disagree nor agree”), forcing respondents onto one of two polar directions. We also adapted individual items by changing their wording, from [transgender] “men” or “women” to “adolescent males” or “adolescent females”.

Secondary outcomes included, first, changes in each of the three T-KAB subscale scores. Second, ratings of warmth toward: a) friends or acquaintances; b) teachers or fellow students; and c) family members. Based on a feelings thermometer originally developed to gauge heterosexuals’ attitudes toward transgender people [18], the thermometers’ scale ranges from 0° to 100° in response to the following prompt: “Think of an imaginary thermometer. The warmer or more favorable you feel toward the [TGD] group in question, the higher the number you should give. The colder or less favorable you feel, the lower the number. If you feel neither warm nor cold toward the group, rate it 50.” Third, we compared responses between PRE and POST to five knowledge questions, each of which had a single correct answer and three distracting alternatives. We classified participants as “fully accepting” if they reached a ceiling temperature of 100° on at least one of the three thermometers.

### Statistical analysis

We used chi square (χ^2^) and one-way analysis of variance (ANOVA) to compare categorical and continuous demographic characteristics at baseline, respectively. We used the McNemar test to compare correct answers in knowledge items between PRE and POST, and the Cochran-Mantel-Haenszel [CMH] χ^2^ to compare categorical variables stratified across interventions. For our primary and secondary outcomes, we used general linear model (GLM) variations: 1) multiple ANOVA (MANOVA) for the seven outcome measures across two intervention types (VIDEO, NO VIDEO) and three time points (PRE, POST, 30-day FOLLOW-UP) and; 2) repeated measures ANOVA to depict change over time using estimated marginal means; and 4) general estimation equations (GEE) to calculate Wald χ^2 ^*p* values comparing the difference between marginal means over time while adjusting for subjects lost to follow up at 30 days. We compared PRE and POST continuous outcomes using paired Student t tests. We repeated the same approach for item-level comparisons, except for an adjustment in significance threshold (from < 0.05 to < 0.01) to account for multiple comparisons.

## Results

**Fig. 1:**
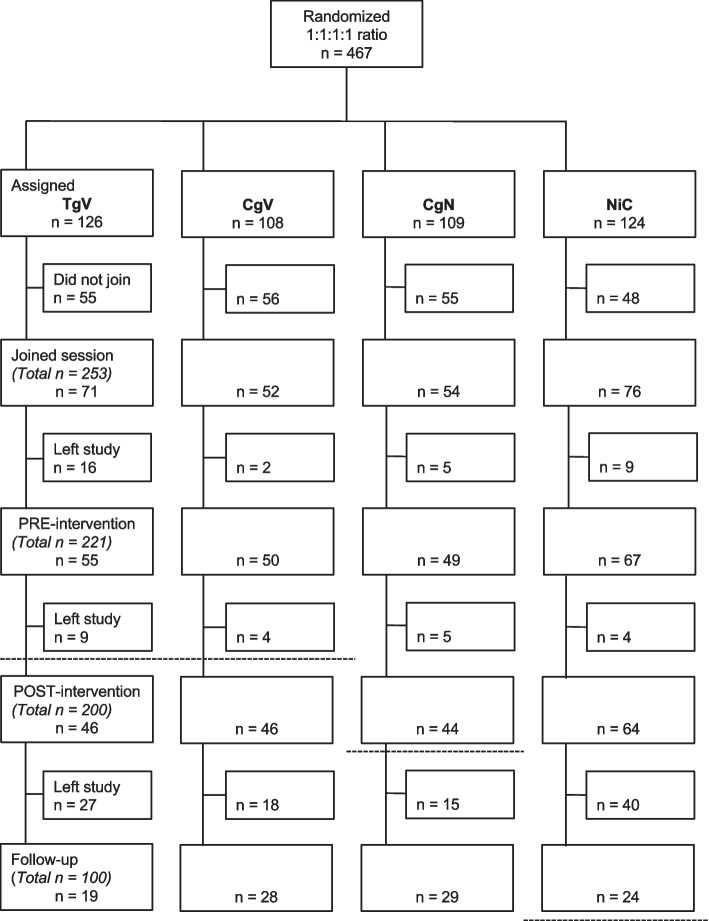
Study flowchart. TgV = transgender woman presenter using embedded videos; CgV = cisgender woman presenter using embedded videos; CgN = cisgender woman presenter using no embedded videos; NiC = no intervention control. TgV + CgV groups = VIDEO intervention; CgN + NiC groups = NO VIDEO control. Dashed lines indicate timepoints at which participants saw the videos

We recruited and proportionally randomized 467 individuals to one of four groups: TgV (*n* = 126), CgV (*N* = 108), CgN (*n* = 109), and NiC (*n* = 124); i.e., 234 to the VIDEO and 233 to the NO VIDEO conditions. As depicted in Fig. 1, 253 (53%) of randomized participants joined their assigned session, 221 (87%) of whom completed assessments at PRE, 200 (79%) at POST, and 100 (40%) at 30-day follow-up. Demographic characteristics did not differ across the four groups (Cochran-Mantel-Haenszel [CMH] |^2^ = 3.42, df = 3, *p* > 0.05, Table 1). Most participants (113; 62%) were involved in the health care professions, as either trainees or practitioners.

**Table 1:**
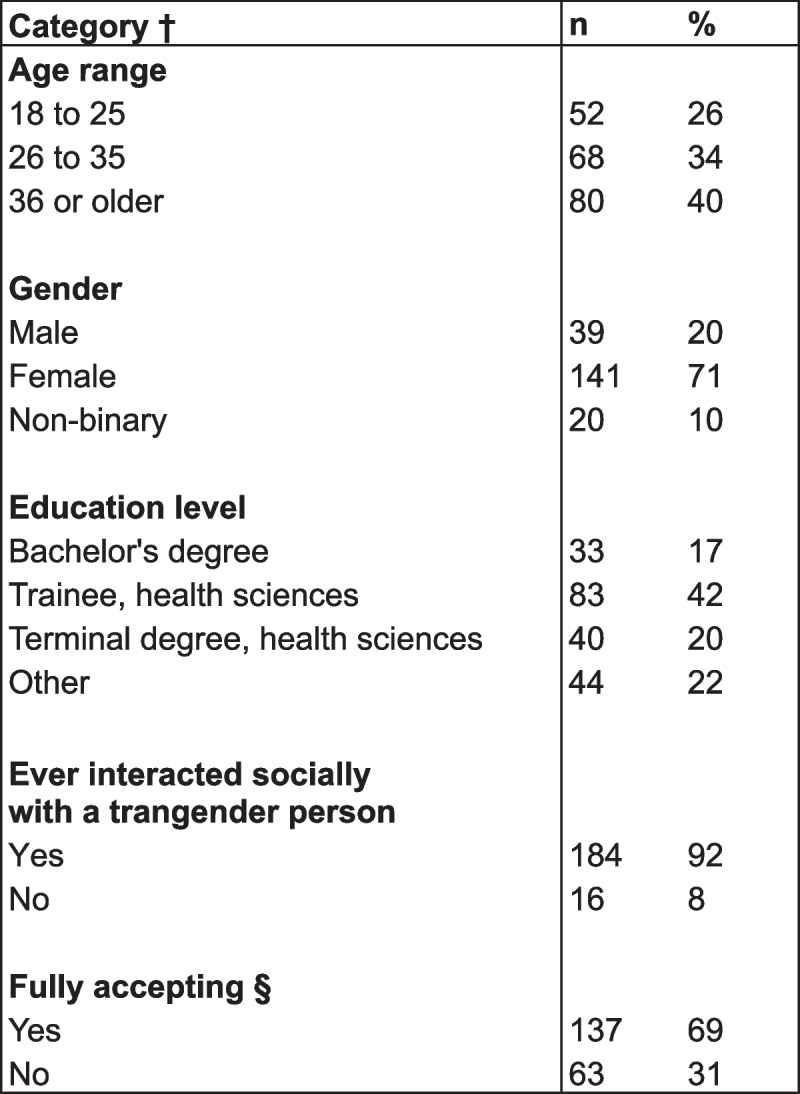
Sample demographics (*n* = 200)

^a^Cochran-Mantel-Haenszel (CMH) across groups > 0.05 for all categories

^b^Defined as having ceiling-level transgender attitude ratings at baseline

**Fig. 2:**
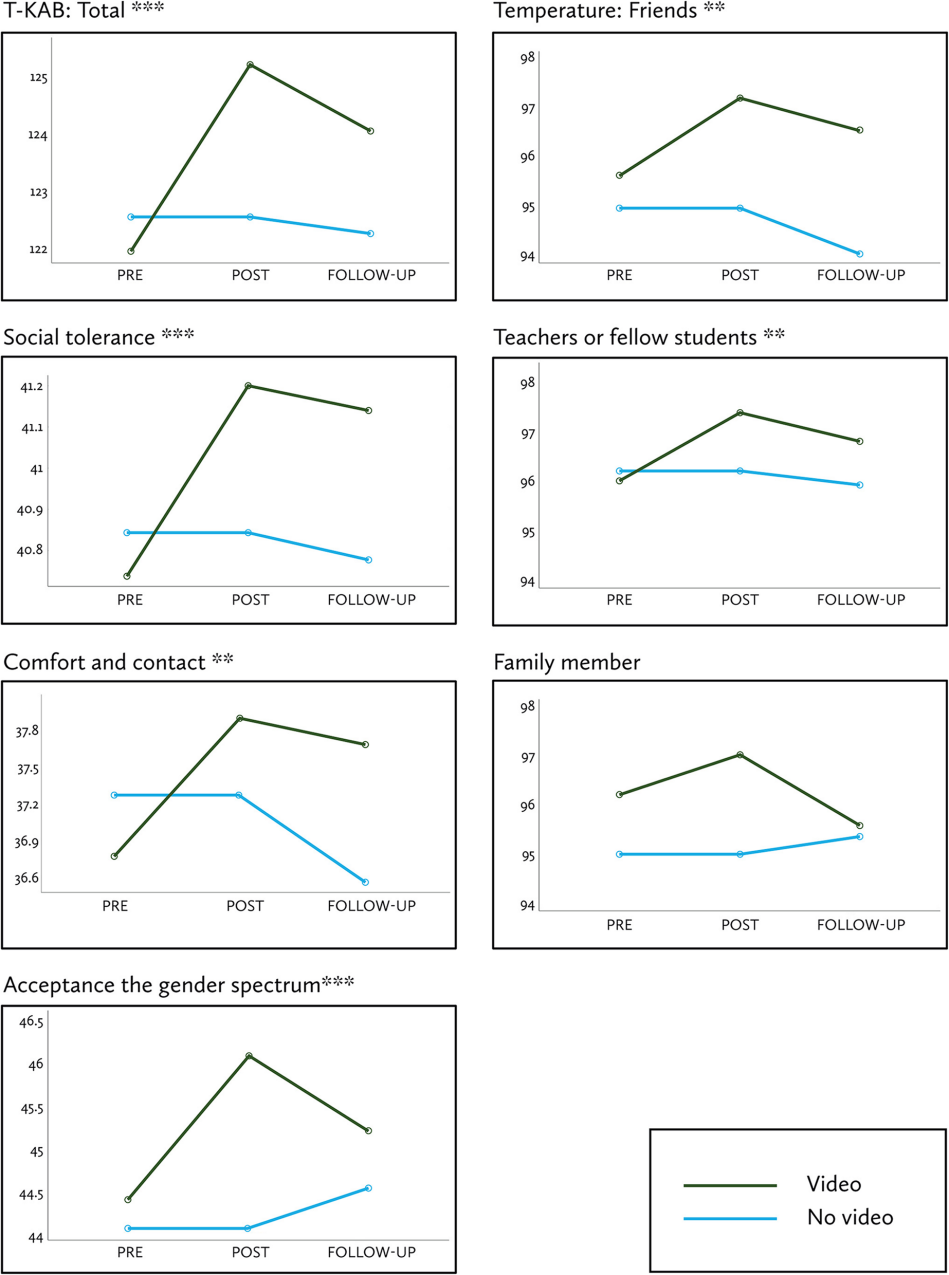
Outcome measure estimated marginal means over time (PRE, POST, 30-day FOLLOW-UP); differences between groups (VIDEO vs NO VIDEO) calculated with general estimation equations (GEE) Wald χ^2^ tests to adjust for subjects lost to follow up at 30 days. * *p* < 0.05. *** *p* < 0.001

Comparing the VIDEO and NO VIDEO groups, we found a group-by-time interaction using 2 × 3 MANOVA for the seven outcome measures (Wilks’ lambda F = 604.54, df = 14, 962, *p* < 0.001.) Mean scores for six of the seven outcome measures increased from PRE to POST in the VIDEO compared to the NO VIDEO group, with an attenuation in response after 30 days (Wald χ^2^ ≥ 14.22, df = 4, *p* ≤ 0.007). The only measure no longer different at the 30-day mark was warmth for a TGD family member (Wald χ^2^ = 7.36, df = 4, *p* = 0.118, ns, Fig. 2).

When combining the three active interventions into a single group, every outcome measure improved between PRE and POST (paired t ≥ 0.59, *p* ≤ 0.02). However, one-way ANOVA revealed no differences across the three active arms (*p* > 0.05), except for the comfort and contact subscale (F = 3.28, *p* = 0.04). When considered separately, each of the groups showed a different level of change after their respective intervention: for six outcome measures in TgV, three in CgV, and two in CgN (Table 2). We provide item-level results in Supplemental Table 1.

**Table 2:**
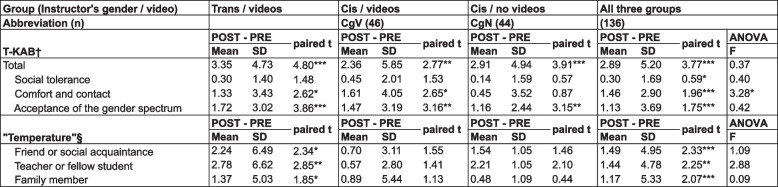
Primary outcomes across active intervention groups

^a^Transgender Knowledge, Attitudes, and Beliefs (T-KAB) Scale: higher values represent more accepting / less trasphobic attitudes (Clark & Hughto, 2020) [17]

^b^Responses to the prompt “Using a scale from zero to 100, please tell us how comfortable you would be interacting with a transgender or non-binary individual as your…” (Modeled after Norton and Herek, 2013 [18])

*p < 0.05

**p < 0.01

***p < 0.001

**Fig. 3:**
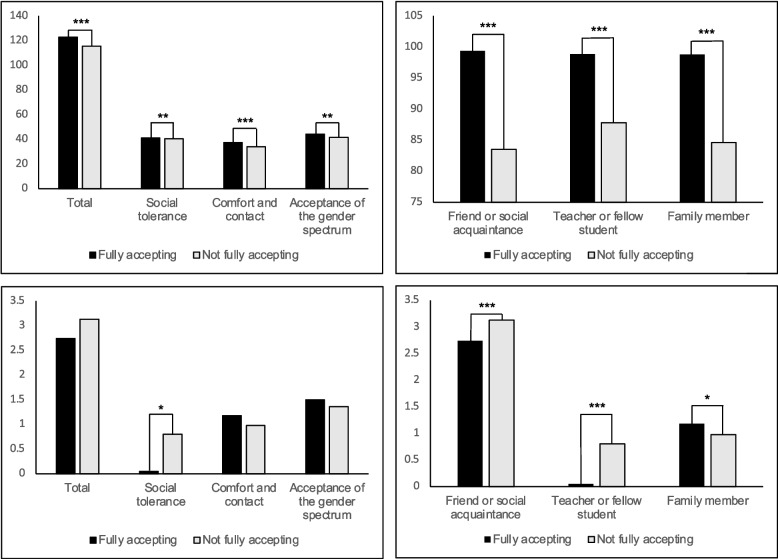
Upper panels depict differences at baseline on the Transgender Knowledge, Attitudes, and Beliefs Scale (T-KAB; range: 22–132); lower panels depict change in T-KAB before and after the intervention (PRE vs POST) in the VIDEO group only. ** *p* < 0.01. *** *p* < 0.001

We compared the response pattern between participants classified as fully accepting (*n* = 137, 69%) and those who were not (*n* = 63, 31%). Those who were fully accepting had higher outcome values at baseline when compared to those who were not (*p* ≤ 0.01 for all, Fig. 3). Mean score changes after the intervention were greater in the non-fully accepting group. For the T-KAB, only the social tolerance scale changed (*p* < 0.05). All three temperature ratings changed from baseline, increasing for friends and teachers (*p* < 0.001), but decreasing for family members (*p* < 0.05).

The increase in participants’ performance on knowledge-based questions before and after the intervention was similar across the three intervention arms (CMH χ^2^ = 2.31, df = 2, *p* > 0.05). Accuracy in pooled response rates improved by a median of 28% (range, 15–51, Table 3).

**Table 3:**
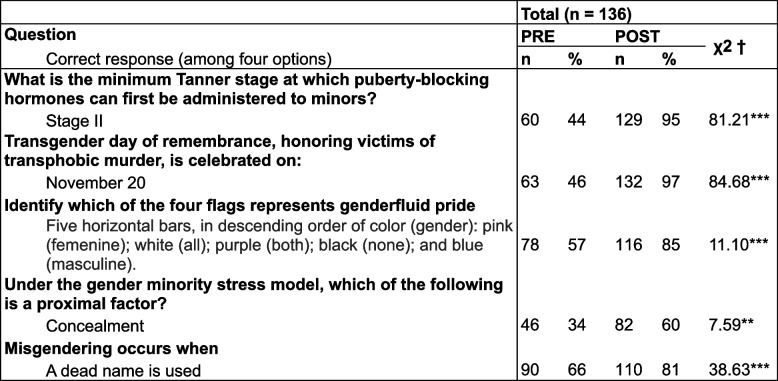
Knowledge items across active intervention groups

^a^McNemar; Cochran-Mantel-Haenszel (CMH) across groups > 0.05 for all questions

**p < 0.01

***p < 001

## Discussion

In this study we found that a didactic enhanced by video clips of two transgender adolescents sharing their living experiences improved knowledge, attitudes, and preconceived misconceptions regarding TGD youth. We found these effects to be effective immediately after the intervention, a finding that may be explained solely based on immediate recall. Importantly, benefits persisted after 30 days. The three interventions we used, once combined into a single video-enhanced group, robustly outperformed the outcomes of the no-intervention control group. We found that instruction by a transgender woman resulted in stronger within-group improvement in outcomes, i.e., before and after the intervention. However, the effect sizes specific to the transgender presenter did not yield between-group differences when compared to the outcomes achieved by a cisgender presenter using the same didactic materials. This is a counterintuitive finding we had not anticipated in our initial hypothesis (H_a_: TgV > CgV).

Our actual results, which support the null hypothesis (Ho: TgV = CgV), are a welcome alternative. Specifically, they provide empirical evidence that a well-informed presenter, regardless of gender, can achieve similar outcomes when using the same didactic resource. In other words, a transgender instructor is not necessary to realize the aims of the educational session. This is important for at least three reasons. First, it can be challenging to find a transgender instructor, particularly in smaller or less welcoming communities. Second, it does not place the responsibility of transgender health education solely on TGD individuals. Finally, it may lead to a virtuous cycle in which cisgender individuals or other LGBTQ+ allies can help expand diffusion of knowledge critical to the acceptance of the gender-diverse community and the individuals who comprise it.

At the time of recording, the two adolescent protagonists were living in a largely comfortable stretch of their gender journey. As such, they did not reflect TGD youth who may be more actively struggling with internalized transphobia or personal non-acceptance. We also note that both protagonists where white, which limits the generalizability of our findings to groups with intersectional identities who are at higher risk of bullying, aggression, or violence [19, 20]. In particular, transgender Black adolescent females and women have been victims of abuse, including of a record-breaking 57 murders in 2021 [21].

Social desirability [22, 23] and stated preference [24] biases are likely to have played a role in the study, as suggested by changes in warmth over time. Specifically, participants’ increases in temperature endured after 30 days, but only with respect to their friends, teachers, or fellow students. By contrast, when pertaining to family members, participants returned to their baseline ratings. This family-specific regression to the mean suggests that subjects may have been less accepting and affirming in their views about those they are most emotionally invested in. At least part of the initial improvements may have had to do more with respondents’ recognition that their attitudes were being studied than with actual change in perceptions, a classic example of the Hawthorne effect, in which behavior changes in response to the awareness of being under observation.

In prior work we had proven the efficacy of brief videos (< 110 s) to reduce depression-related stigma and increase treatment-seeking intentions among white [25], Black [26], and transgender [27] protagonists of a similar age. The active component common across the three studies was an adolescent sharing their personal narrative in an emotionally legitimate manner that highlighted improvements related to acceptance (of racial or gender diversity) and/or treatment (for depression and attendant suicidal ideation). The impact of videos that depict the shared living experience of TGD youth can partly be explained through the contact exposure theory [28], which describes how the favorable interaction between members of historically divided communities can improve mutually stigmatized perceptions. This putative mechanism is supported by the mere exposure phenomenon [29, 30] through which repeated exposure to a novel stimulus can be sufficient to improve attitudes and enhance appreciation of the stimulus.

Having proven the efficacy of short videos in three separate RCTs, each one using a large, crowdsourced sample of over one thousand participants, we sought in this new study ways of applying similar video-based components into healthcare education. We were interested in finding optimal ways to incorporate this content in the training of medical and mental health trainees and professionals, as well as of staff supporting clinical settings. From the outset, our intention was to also include school and other youth-facing environments that can be rife with misunderstanding, non-acceptance, and transphobia. Similarly, we were keen to reach individuals who may mean well but not have the right knowledge or skills to interact effectively with TGD youth. In using materials that are brief and readily available for download free of charge, we have sought in this, as in prior studies [31–34] to reduce the burden on new instructors seeking to develop evidence-based and up-to-date training resources. We anticipate—and indeed encourage—educators to adapt these video-enhanced materials for goals specific to the needs of their group of learners.

### Limitations

Our study has several noteworthy limitations, which may inform future refinements. First, most of the enrolled participants were already supportive and well-versed in TGD health. We classified more than two thirds of the study’s participants as “fully accepting”. Their ceiling-level baseline ratings limited possible improvement in perceptions. Contributing to this imbalance was a lower than anticipated participation of undergraduate students and others not yet committed to the healthcare professions. And yet, our ability to document immediate and sustained attitude improvements after a brief intervention, even among this skewed group, suggests the considerable potential for impact among those with limited knowledge or exposure to TGD youth. As a point reference, it is estimated that less than half of Americans (42%) personally know someone who is transgender [35]. Second, the dropout rate was significant, particularly the 50% completion rate at the 30-day follow-up. Third, we cannot rule out the possibility of insufficient power to find differences across the three intervention groups. We consider this an unlikely possibility, given that a power calculation based on our main outcome (Cohen’s d = 0.67) suggests that a sample of 36 participants per group (VIDEO vs NO VIDEO) would have been sufficient to detect a difference on the T-KAB with 80% power and an alpha level of 0.05 [36]. Fourth, we recognize having insufficient power to evaluate differences across the participants’ four education categories, limiting our generalizability to specific groups, such as a lay audience or specific types of professionals. Further research could evaluate group- or learning-goal-specific adaptations of curricular or video content. Fifth, even when considering the sustained yet attenuated improvements after 30 days, we can’t assume changes will be sustained over longer periods of time. Repeated interventions, as those of a spiral curriculum, may be needed to sustain enduring change. Sixth, by featuring two transgender youth on the male / female binary, we did not adequately represent adolescents who are gender queer, gender fluid, or otherwise nonbinary. Similarly, our study did not include the intersectionality [37] of transgender youth of color, or those with additional clinical needs, such as autism spectrum disorders. Finally, we cannot claim that short-term change in perceptions and knowledge will translate into enhancements in clinical practice or other routine interactions with TGD youth, as those in schools or sports teams. Similarly, we cannot assume longer-term impact, as could be represented by active engagement in political support or advocacy efforts. Co-partnering between the LGBTQ+ community and its allies is particularly urgent in response to the recent resurgence of official, state- sanctioned transphobic policies, including the criminalization of potentially life-saving gender-affirming treatment.

### Implications and contribution

This evidence-based and empirically validated educational resource can improve knowledge about and acceptance of TGD youth. The healthcare trainees and professionals these materials were originally intended for are not their exclusive beneficiaries. Beyond healthcare, any youth-facing environment stands to gain from them, as in educational, sports, legal, political, or housing interfaces.

### Next steps

Disseminating an educational initiative such as this, especially if embedded into existing curricula as early as grade school, could have enduring effects. Schools, sports teams, or any other setting in which TGD youth need to live and navigate—sometimes against a hostile current—could use educational interventions such as this. Finally, one promising alternative to expanding the reach of video-based interventions to reduce transphobia is through social media platforms. We are encouraged by our initial demonstration in two non-inferiority RCTs of the equivalence between professionally filmed and edited materials such as the ones in this study and self-made, shorter alternatives that can be directly uploaded to the web through applications such as TikTok or Instagram [38].

## Supplementary Information

**Additional file 1: Supplemental Table 1.** Transgender Knowledge, Attitudes, and Beliefs (T-KAB): subscales and component items.

## Data Availability

The datasets generated during and/or analysed during the current study are available in the QUALab repository of the Yale Child Study Center, at https://tinyurl.com/3y879v93.
