# Supplementary material for: Collaborating with transgender youth to educate healthcare trainees and professionals: randomized controlled trial of a didactic enhanced by brief videos
Source: BMC Public Health. 2022 Dec 26;22:2427. doi: 10.1186/s12889-022-14791-5 (PMC9791767; doi:10.1186/s12889-022-14791-5)
Supplement: Supplementary file 1 — Additional file 1: Supplemental Table 1. Transgender Knowledge, Attitudes, and Beliefs (T-KAB): subscales and component items. [file 12889_2022_14791_MOESM1_ESM.pdf]

SUPPLEMENTAL TABLE 1
Transgender Knowledge, Attitudes, and Beliefs (T-KAB): subscales and component items

**Subscale** / component items†	TgV (n = 46)			CgV (n = 46)			CgN (n = 44)			All (n = 136)		
	PRE - POST		t	PRE - POST		t	PRE - POST		t	PRE - POST		t
	Mean	SD		Mean	SD		Mean	SD		Mean	SD	
**Social Tolerance (ST)**												
1 Transgender people should have the opportunity to undergo operations to change their anatomy	0.13	0.34	2.60**	0.06	0.32	1.35	0.00	0.50	0.00	0.07	0.61	1.26
2 Transgender people should be accepted completely into our society	0.04	0.36	0.81	0.09	0.78	0.75	0.27	0.39	0.27	0.06	0.80	0.86
3 Transgender people should have the opportunity to change their name	0.11	0.43	1.70*	0.30	1.04	1.96*	2.35	0.01	2.35*	0.18	0.69	2.99**
4 Organizations that promote transgender rights are necessary	0.02	0.33	0.44	-0.02	0.33	-0.44	1.67	0.05	1.67	0.03	0.34	1.00
5 It should be illegal for people to have their genitalia surgically altered (R)	0.09	0.78	0.75	-0.02	0.90	-0.16	-1.95	0.03	-1.95*	-0.07	0.73	-1.18
6 Being transgender is a sin (R)	0.07	0.44	1.00	-0.02	0.71	-0.21	0.00	0.50	0.00	0.02	0.49	0.35
7 Transgender people are a worthwhile part of our society	0.02	0.15	1.00	0.06	0.25	1.77*	0.00	0.30	0.00	0.03	0.21	1.64
**Comfort and contact (CC)**												
1 I avoid interacting with people whose gender is unclear to me (R)	0.04	0.92	0.32	0.00	0.47	0.00	-0.31	0.38	-0.31	0.00	0.82	0.00
2 People with both breasts and a penis make me uncomfortable (R)	0.24	1.20	1.36	0.00	0.75	0.00	0.66	0.26	0.66	0.11	0.97	1.32
3 When I meet someone, it is important for me to be able to identify them as a man or a woman (R)	0.48	0.69	4.70***	0.26	0.71	2.48**	1.35	0.09	1.35	0.32	0.86	4.39***
4 I feel uncomfortable when I cannot tell if someone is a man or a woman (R)	0.35	1.02	2.32*	0.34	0.82	2.86**	3.17	0.00	3.17**	0.39	0.94	4.81***
5 I would feel uncomfortable if a close family member were dating a transgender person (R)	0.11	0.95	0.78	0.09	1.63	0.36	2.56	0.01	2.56**	0.18	1.22	1.75
6 I would feel comfortable if I learned my neighbor is transgender	0.04	2.05	0.14	-0.02	1.74	-0.08	-0.09	0.47	-0.09	0.00	1.84	0.00
7 I would feel comfortable if I learned my neighbor is transgender	0.07	2.00	0.22	-0.21	1.38	-1.06	1.46	0.08	1.46	0.12	1.97	0.70
**Acceptance of the gender spectrum subscale (ACC)**												
1 A person with a vagina can be a man	0.48	1.03	3.16**	0.32	1.02	2.14*	2.49	0.01	2.49**	0.36	0.94	4.469**
2 A person with a penis can be a woman	0.33	0.60	3.70***	0.21	0.66	2.22*	0.78	0.22	0.78	0.22	0.76	3.34***
3 Female-to-male transgender people are real men	0.20	0.45	2.93**	0.13	1.14	0.77	1.23	0.11	1.23	0.15	0.82	2.18*
4 Male-to-female transgender people are real women	0.13	0.45	1.96*	0.11	1.11	0.66	2.21	0.02	2.21*	0.13	0.74	1.98*
5 A person transitioning from female to male should be able to use a men's bathroom	0.17	0.49	2.43*	0.15	0.47	2.20*	1.35	0.09	1.35	0.13	0.44	3.54***
6 There are only two genders: male and female (R)	0.20	0.62	2.14*	0.21	0.46	3.15**	2.46	0.01	2.46**	0.19	0.51	4.38***
7 Sex and gender are the same thing (R)	0.07	1.41	0.32	0.23	0.60	2.69**	2.20	0.02	2.20*	0.15	0.93	1.95*
8 A person transitioning from male to female should be able to use a women's bathroom	0.15	0.47	2.20*	0.11	0.43	1.70*	1.95	0.03	1.95	0.13	0.43	3.34***

† Higher scores indicate greater knowledge and more accepting attitudes/beliefs towards transgender individuals. Items are scored on a 6-point Likert scale ranging from 1 ("strongly disagree") to 6 ("strongly agree"). (R) denotes reverse-scored items (Clark & Hughto, 2020)

* $p < 0.05$
** $p < 0.01$
*** $p < 0.001$
